# The Altmetrics Collection

**DOI:** 10.1371/journal.pone.0048753

**Published:** 2012-11-01

**Authors:** Jason Priem, Paul Groth, Dario Taraborelli

**Affiliations:** 1 School of Information & Library Science, University of North Carolina at Chapel Hill, Chapel Hill, North Carolina, United States of America; 2 Department of Computer Science and The Network Institute, VU University Amsterdam, Amsterdam, The Netherlands; 3 The Wikimedia Foundation, San Francisco, California, United States of America; The Centre for Research and Technology, Hellas, Greece

## Introduction

What paper should I read next? Who should I talk to at a conference? Which research group should get this grant? Researchers and funders alike must make daily judgments on how to best spend their limited time and money–judgments that are becoming increasingly difficult as the volume of scholarly communication increases. Not only does the number of scholarly papers continue to grow, it is joined by new forms of communication from data publications to microblog posts.

To deal with incoming information, scholars have always relied upon filters. At first these filters were manually compiled compendia and corpora of the literature. But by the mid-20th century, filters built on manual indexing began to break under the weight of booming postwar science production. Garfield [1] and others pioneered a solution: automated filters that leveraged scientists own impact judgments, aggregating citations as “pellets of peer recognition.” [2].

These citation-based filters have dramatically grown in importance and have become the tenet of how research impact is measured. But, like manual indexing 60 years ago, they may today be failing to keep up with the literature’s growing volume, velocity, and diversity [3].

Citations are heavily gamed [4]–[6] and are painfully slow to accumulate [7], and overlook increasingly important societal and clinical impacts [8]. Most importantly, they overlook new scholarly forms like datasets, software, and research blogs that fall outside of the scope of citable research objects. In sum, citations only reflect *formal acknowledgment* and thus they provide only a partial picture of the science system [9]. Scholars may discuss, annotate, recommend, refute, comment, read, and teach a new finding before it ever appears in the formal citation registry. We need new mechanisms to create a subtler, higher-resolution picture of the science system.

### The Quest for Better Filters

The scientometrics community has not been blind to the limitations of citation measures, and has collectively proposed methods to gather evidence of broader impacts and provide more detail about the science system: tracking acknowledgements [10], patents [11], mentorships [12], news articles [8], usage in syllabuses [13], and many others, separately and in various combinations [14]. The emergence of the Web, a “nutrient-rich space for scholars” [15], has held particular promise for new filters and lenses on scholarly output. Webometrics researchers have uncovered evidence of informal impact by examining networks of hyperlinks and mentions on the broader Web [16]–[18]. An important strand of webometrics has also examined the properties of article download data [7], [19], [20].

The last several years, however, have presented a promising new approach to gathering fine-grained impact data: tracking large-scale activity around scholarly products in online tools and environments. These tools and environments include, among others:

- social media like *Twitter* and *Facebook*
- online reference managers like *CiteULike*, *Zotero*, and *Mendeley*
- collaborative encyclopedias like *Wikipedia*
- blogs, both scholarly and general-audience
- scholarly social networks, like *ResearchGate* or *Academia.edu*
- conference organization sites like *Lanyrd.com*

Growing numbers of scholars are using these and similar tools to mediate their interaction with the literature. In doing so, they are leaving valuable tracks behind them–tracks with potential to show informal paths of influence with unprecedented speed and resolution. Many of these tools offer open APIs, supporting large-scale, automated mining of online activities and conversations around research objects [21].

Altmetrics [22], [23] is the study and use of scholarly impact measures based on activity in online tools and environments. The term has also been used to describe the metrics themselves–one could propose in plural a “set of new altmetrics.” Altmetrics is in most cases a subset of both scientometrics and webometrics; it is a subset of the latter in that it focuses more narrowly on scholarly influence as measured in online *tools and environments,* rather than on the Web more generally.

Altmetrics may support finer-grained maps of science, broader and more equitable evaluations, and improvements to the peer-review system [24]. On the other hand, the use and development of altmetrics should be pursued with appropriate scientific caution. Altmetrics may face attempts at manipulation similar to what Google must deal with in web search ranking. Addressing such manipulation may, in-turn, impact the transparency of altmetrics. New and complex measures may distort our picture of the science system if not rigorously assessed and correctly understood. Finally, altmetrics may promote an evaluation system for scholarship that many argue has become overly focused on metrics.

### Scope of this Collection

The goal of this collection is to gather an emerging body of research for the further study and use of altmetrics. We believe it is greatly needed, as important questions regarding altmetrics’ prevalence, validity, distribution, and reliability remain incompletely answered. Importantly, the present collection, which has the virtue of being online and open access, allows altmetrics researchers to experiment on themselves.

The collection’s scope includes:

- Statistical analysis of altmetrics data sources, and comparisons to established sources
- Metric validation, and identification of biases in measurements
- Validation of models of scientific discovery/recommendation based on altmetrics
- Qualitative research describing the scholarly use of online tools and environments
- Empirically-supported theory guiding altmetrics’ use
- Other research relating to scholarly impact in online tools and environments.

The current collection includes articles that address many of these areas. It will publish new research on an ongoing basis, and we hope to see additional contributions appear in the coming months. We look forward to building a foundation of early research to support this new field.
